# Higher levels of eco-distress in psychotherapy out-patients with depressive and anxious symptoms are predicted by emotion regulation strategies

**DOI:** 10.3389/fpsyt.2025.1664040

**Published:** 2025-10-30

**Authors:** Nadja Gebhardt, Jil Beckord, Alexander Bäuerle, Martin Teufel, Hans-Christoph Friederich, Christoph Nikendei

**Affiliations:** ^1^ Department of General Internal Medicine and Psychosomatics, Heidelberg University Hospital, Heidelberg, Germany; ^2^ Clinic for Psychosomatic Medicine and Psychotherapy, University of Duisburg Essen, LVR-University Hospital Essen, Essen, Germany; ^3^ Center for Translational Neuro- and Behavioral Sciences (C-TNBS), University of Duisburg Essen, Essen, Germany; ^4^ DZPG (German Centre for Mental Health – Partner Site Heidelberg/Mannheim/Ulm), Heidelberg, Germany

**Keywords:** eco-distress, eco-anxiety, climate anxiety, anxiety, depression, psychometric assessment, emotion regulation strategies

## Abstract

**Background:**

Psychotherapy patients are particularly vulnerable to the experience of eco-distress, often referred to as climate anxiety or eco-anxiety. Eco-distress can foster pro-environmental behavior, but its various symptoms might as well be functionally impairing and are negatively correlated with psychological well-being. The link between eco-distress and depressive and anxiety symptoms, as well as the use of dysfunctional emotion regulation strategies, may explain this vulnerability and suggest ways to promote resilience.

**Methods:**

Psychotherapy out-patients were screened at T1 (*n* = 203) and again five months later (T2; *n* = 79) for anxious (Generalized Anxiety Disorder Scale; GAD-7) and depressive symptoms (Patient Health Questionnaire; PHQ-9) and for eco-distress (Eco-Anxiety Questionnaire, EAQ-22; Generalized Anxiety Disorder Scale-Climate Version; GAD-7-C; Climate Change-Man-Made Disaster Distress Scale; CC-MMDS). Emotion regulation strategies were assessed at T1. Factorial validity was tested for eco-distress questionnaires. The relationship of eco-distress, depressive and anxious symptoms, and emotion regulation strategies was tested via multivariate models, multiple regression analysis, and mediation analysis.

**Results:**

The EAQ-22 and GAD-7-C showed good model fit, the factorial structure of the CC-MMDS had to be adapted. Participants who screened positive for a generalized anxiety disorder and/or a depressive disorder at T1 reported higher levels of eco-distress, but changes in anxious or depressive symptoms from T1 to T2 did not predict a change in eco-distress. At T1, *Rumination* and *Catastrophizing* predicted higher scores of eco-distress for all three questionnaires. However, emotion regulation strategies did not mediate the effect of depressive and anxious symptoms on eco-distress.

**Conclusion:**

Eco-distress is associated with the frequent use of the emotion regulation strategies *Catastrophizing* and *Rumination* and is higher in individuals with depressive and anxious symptoms. Addressing the use of these emotion regulation strategies in individuals could promote psychological resilience when facing the climate crisis.

## 1 Introduction

Climate change is one of the environmental crises caused by humans, which increases the imbalance of the Earth’s system as a whole (1). It interacts with other environmental crises caused by human influence, such as ocean acidification, air pollution, and the destruction of eco-systems. Thereby, climate change and other ecological crises are a severe threat to the basis of human livelihood (2). Mitigation and adaption efforts remain inadequate despite the accumulating knowledge about the multi-layered risks for our physical and mental health resulting from a rapidly changing climate with increasing temperatures, sea level rise, more heat waves, droughts, floods, and sand or dust storms (3).

The knowledge about human-induced environmental degradation and the anticipation of its future consequences can induce psychological distress in individuals (4, 5). This distress can express itself through a range of negative emotions, such as anxiety, sadness, guilt, despair, grief, or anger (6). It can be accompanied by cognitive indicators such as difficulty concentrating or fatigue, physiological indicators such as muscle tension or nausea, and behavioral indicators, such as poor sleep, constant alertness, or social withdrawal (7–9). While these reactions are oftentimes subsumed under the terms climate anxiety or eco-anxiety (5, 10), we will use the broader term of eco-distress to account for the fact that the concept encompasses other emotions than anxiety, as well as cognitive and physical impacts (11). With ongoing climate change, the prevalence of eco-distress is expected to rise further, especially in younger generations (8).

Although eco-distress is considered an adequate response to the threat posed by climate change and can foster pro-environmental behavior, it also shows a negative correlation with well-being and can lead to functional impairments in its extreme forms (12–16). Functional impairments can be understood as behaviors, emotions, thoughts, and physical symptoms limiting individuals in personal and occupational spheres of life, which can cause severe losses in quality of life on a personal level and substantial economic losses on a societal level (17–19). Most eco-distress questionnaires assess both negative emotional reactions, as well as different aspects of resulting functional impairments (20). Functional impairments due to eco-distress may be very similar or identical to those of depression (e.g. sleep disturbances) or anxiety (e.g. arousal), without fulfilling diagnostic requirements for depressive or anxiety disorders (21). In a previous study, patients with mental health impairments such as depression or anxiety disorders showed a heightened vulnerability for the experience of higher levels of eco-distress (22). The cause and course of the impairing consequences of eco-distress and the heightened vulnerability of persons with co-existing general mental health impairments are not yet understood and warrant further attention to minimize the functional impairments resulting from eco-distress, especially in vulnerable subgroups.

**Figure 1:**
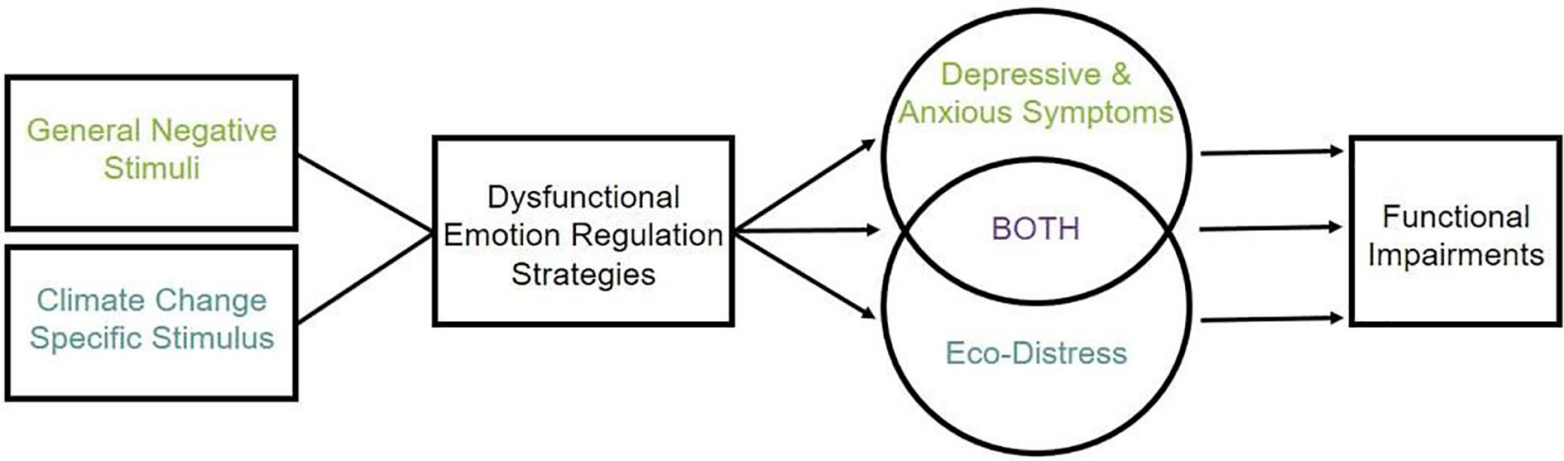
Schematic display of the hypothesized relationship of emotion regulation strategies, eco-distress, depressive and anxious symptoms, and functional impairments.

The framework of appraisal theories offers a possible explanation why the experience of eco-distress is linked to both functional impairments and pro-environmental behavior (23, 24). These theories posit that functional impairments are the result of an intense negative emotional reaction to a stimulus without the capacity to effectively regulate the response to the stimulus. In the context of the climate crisis, individuals who acknowledge the climate crisis as a threat and simultaneously do not have the capacity to effectively regulate the ensuing emotional reaction are prone to exhibit severe levels of eco-distress, accompanied by functional impairments. If, on the other hand, a person acknowledges climate change as a threat and is able to regulate the ensuing emotional response, this might foster pro-environmental behavior and motivate action (see **Figure 1**). Previous research has already addressed emotion regulation strategies and eco-distress in children (25), in relation to pro-environmental behavior (16), and in relation to worry about the future (26). Deepening our understanding of which emotion regulation strategies are relevant when faced with the threat of the climate crisis would be helpful to support people in dealing with this threat in a constructive manner. Moreover, emotion regulation strategies might represent common underlying dysfunctional processes of eco-distress and depressive and anxious symptoms, thereby explaining the heightened vulnerability of persons with mental health impairments.

Our aim was to explore the interplay of eco-distress, depressive and anxious symptoms, and emotion regulation strategies. As persons with co-existing mental health impairments are particularly vulnerable toward the experience of eco-distress, we assessed these aspects in a population of psychotherapy out-patients. No questionnaires have been evaluated regarding their psychometric properties in a clinical population before, thus our first research aim was to establish which questionnaires are suitable to assess eco-distress in a clinical sample. We hypothesized that the questionnaires which had been validated in the general population would show an acceptable fit in a clinical population, as well (H1). In a second step, we explored how elevated levels of depressive and anxious symptoms interact with eco-distress, both cross-sectionally and longitudinally. We hypothesized that psychotherapy out-patients with a pronounced anxious and depressive symptomology would show higher scores of eco-anxiety (H2). Finally, we were interested in identifying which emotion regulation strategies are associated with eco-distress and whether they mediate the relationship of depressive and anxious symptoms with eco-distress. As there was no previous literature on this relationship, this hypothesis was tested exploratory and not directed (H3).

## 2 Methods

The presentation of our analysis is structured according to the STROBE guidelines for cross-sectional studies (27). The study was approved by the ethics committee of the Medical Faculty of the University of Heidelberg (S-249/2023) and is in line with the Declaration of Helsinki. The design was pre-registered at Open Science Framework (https://osf.io/zgrqe/?view_only=d75a73f2e1b547aa8d1eeff00fc5323f).

### 2.1 Participants and procedure

Participants were psychotherapy out-patients at the Heidelberg Institute for Psychotherapy (HIP), Heidelberg, Germany, recruited between 09.10.2023 and 17.09.2024. The HIP is a training institute for psychotherapy with a psychodynamic and a systemic focus (28). At the HIP, both medical and psychological psychotherapists in training offer supervised out-patient psychotherapy to clients diagnosed with depressive disorders, anxious disorders, stress-related disorders, and personality disorders. If patients agreed to participate, they filled out the questionnaire in paper or online and had the option to indicate an e-mail address to be contacted again for a re-assessment five months later. Participants were eligible if they were 18 years or older, already in psychotherapy treatment at the HIP or about to start, and capable of giving informed consent. Patients’ psychiatric diagnoses were not part of our data set. Analyses based on categorizations of participants into groups with and without a probable diagnosis of generalized anxiety disorder or depression were thus solely based on psychometric testing. The focus of our study was the assumed general heightened vulnerability of psychotherapy patients, not the relationship of eco-distress and specific diagnoses. We assessed symptoms of depression and anxiety because they are common features of eco-distress questionnaires and thus likely part of the explanation for the association of eco distress and general mental health impairments. All participants were informed about the study’s procedure and gave written informed consent to participate.

### 2.2 Measures

#### 2.2.1 Eco-distress questionnaires

As the definition of eco-distress differs between questionnaires, we decided to employ several questionnaires covering different aspects of eco-distress which had already been successfully validated in the general German-speaking population:

The Eco-Anxiety Questionnaire (EAQ-22) (29) was first developed in Hungary and its factorial structure has successfully been replicated in a German sample (30). It consists of two subscales: *habitual ecological worry*, which encompasses climate-change related negative emotional reactions; and *negative consequences of eco-anxiety*, which encompasses functional impairments through climate-change related thoughts and emotions, such as poor sleep, constant alertness, or muscle tension.

The Generalized Anxiety Disorder Scale – Climate Version (GAD-7-C) (31) is an adaption of the Generalized Anxiety Disorder Scale (GAD-7) (32), adding the specification “…when thinking about climate change” to the items assessing symptoms of generalized anxiety disorder. Although this questionnaire has already been used in a clinical sample (22), data on its factorial validity has not yet been published.

The Climate Change Version of the Man Made Disaster-Related Distress Scale (CC-MMDS) (33) is an adaption of the Man Made Disaster-Related Distress Scale (34) which has originally been developed to assess psychological distress after man-made disasters. The CC-MMDS defines climate change as a man-made disaster and consists of two subscales: *Psychological Distress*, which encompasses climate change-related emotional reactions and functional impairments; and *Change of Existing Belief Systems*, which assesses whether society’s handling of climate change affects people’s general beliefs about society, politics, and the future.

#### 2.2.2 Depressive and anxious symptoms

Anxious symptoms were assessed with the General Anxiety Disorder Scale (GAD-7) in its original form (32), depressive symptoms were assessed with the Patient Health Questionnaire (PHQ-9) (35). Both instruments are well established in mental health research and cut-off scores have been established to screen for depressive disorders or generalized anxiety disorders and to assess severity of anxious or depressive symptoms. Regarding established cut-off scores for GAD-7 and PHQ-9, participants screened positive for a generalized anxiety disorder if GAD-7 ≥ 10. In a sample of *n* = 2,740 patients, this cut-off showed a sensitivity of 89% and a specificity of 82% (32). For the PHQ-9, participants screened positive for a depressive disorder if PHQ-9 ≥ 10. In a sample of *n* = 6,000 patients, this cut-off showed a sensitivity of 88% and a specificity of 88% (35).

#### 2.2.3 Emotion regulation strategies

Emotion regulation refers to strategies which change the intensity, duration, and type of an emotional reaction (36). They play an integral role in the development of general mental health impairments (37). The Cognitive Emotion Regulation Questionnaire-Short-Climate Change Version (CERQ-SC) is an adaption of the Emotion Regulation Questionnaire-Short (38), which assesses nine different emotion regulation strategies, such as R*umination*, *Catastrophizing*, or *Acceptance*. It is widely used and has already been applied to study the link of emotion regulation strategies and climate action (16). It was adapted by the research team to address emotion regulation strategies in the context of climate change-related emotions, e.g. *Rumination:* “I think about how I feel because of climate change”. The modified version of the questionnaire in its original and in a translated version is made available on OSF (https://osf.io/zgrqe/?view_only=d75a73f2e1b547aa8d1eeff00fc5323f).

### 2.3 Data analysis

In a first step, model fit of the three eco-distress questionnaires in a clinical sample was tested. We ran confirmatory factor analyses (CFA) using the R package lavaan (39) for eco-distress (EAQ-22, GAD-7-C, and CC-MMDS), employing a maximum likelihood robust (MLR) estimator and full information maximum likelihood estimation for missing values. Model fit was considered acceptable if Comparative Fit Index (CFI) and Tucker Lewis Index (TLI) >.90, a Root Mean Square Error of Approximation (RMSEA) <.08, and a Standardized Root Mean Square Residual (SRMR) <.08 (40, 41). Furthermore, reliability, convergent validity, discriminant validity, and measurement invariance of the EAQ-22, GAD-7-C, and CC-MMDS were tested. Reliability was assessed by calculating Cronbach’s α. Convergent validity was assessed by calculating correlations between the EAQ-22, GAD-7-C, and CC-MMDS. Discriminant validity was assessed by calculating correlations between eco-distress (EAQ-22, GAD-7-C, CC-MMDS) and depressive and anxious symptoms (GAD-7 and PHQ-9), expecting significantly smaller correlations of the eco-distress questionnaires with GAD-7 and PHQ-9 than with other eco-distress questionnaires. Configural, scalar, and metric measurement invariance was assessed for age and gender. Invariance was defined as ΔCFI <.01 and ΔRMSEA <.015 (42, 43).

In a second step, we explored the relationship of depressive and anxious symptoms with eco-distress. Differences in scores on the eco-distress questionnaires for participants who screened positive or negative for a generalized anxiety disorder and/or a depressive disorder were calculated via multivariate models. Furthermore, we invited participants to fill out the eco-distress questionnaires, the GAD-7 and the PHQ-9 again after 5 months. We then calculated change scores with Δ = (*T1* – *T2*), and performed multiple regression analyses with participants’ change scores for depressive and anxious symptoms (PHQ-9 and GAD-7) as predictors for eco-distress change scores.

In a third step, we explored the relationship of emotion regulation strategies with eco-distress and the possible mediating effect of emotion regulation strategies on the association of eco-distress and depressive and anxious symptoms. We performed multiple regression analyses with the nine emotion regulation strategies of the CERQ-SC as predictors for eco-distress to evaluate dysfunctional cognitive processes which could represent possible intervention targets. Finally, we ran mediation analyses with depressive and anxious symptoms as predictors, emotion regulation strategies as mediators, and eco-distress as outcome variable.

## 3 Results

### 3.1 Sample characteristics

By approaching *n* = 319 individuals, *n* = 98 (31%) participants who had been psychotherapy out-patients for *M* = 45.15 weeks [*SD* = 36.2; missing data for *n* = 17 (16%)] could be recruited. Participants were recruited while they were in the waiting area of the institute. Of these, *n* = 44 (22%) filled out the questionnaire online via a link provided by the study team and *n* = 54 (26%) in a paper-pencil version. The completion of the questionnaire took place at home. Participants handed in the paper-pencil versions when showing up for their next appointment. Additionally, *n* = 105 (52%) participants filled out the questionnaire in a paper-pencil version which had been integrated into the regular psychometric evaluation at the beginning of their treatment. In all instances, participation was optional. However, no patients included in our sample skipped any of the questionnaires during the intake evaluation. Thus, the final sample consisted of *n* = 203 participants. Of these, *n* = 127 (63%) identified as female and *n* = 66 (33%) as male, none as other [missing data for *n* = 10 (4%)]. Participants were *M* = 37.08 (*SD* = 13.12) years old. Means, standard deviations, median, and range for all eco-distress and general mental health questionnaires are provided in **Table 1**.

**Table 1:** Means, standard deviations, median, and range for all climate change-related and general mental health questionnaires.

Questionnaire	Mean	SD	MD	Min	Max
PHQ-9	10.02	5.20	10	0	24
GAD-7	8.72	4.30	8	1	21
GAD-7-C	2.50	3.56	1	0	21
EAQ-22-EW	36.95	9.23	38	13	52
EAQ-22-NC	12.18	3.99	11	9	30
CC-MMDS-PD	17.70	10.00	13.5	7	47
CC-MMDS-BS	18.05	9.27	18	5	35
CC-MMDS-PD_OLD	27.36	15.41	22.5	11	73
CC-MMDS-BS_OLD	18.58	9.70	18	5	35

PHQ-9, Patient Health Questionnaire; GAD-7, Generalized Anxiety Disorder Scale; GAD-7-C, Generalized Anxiety Disorder Scale Climate Version; EAQ-22-EW, Eco-Anxiety Questionnaire, subscale “ecological worry”; EAQ-22-NC, Eco-Anxiety Questionnaire, subscale “negative consequences of eco-anxiety”; CC-MMDS-PD, Climate Change Version of the Man Made Disaster-Related Distress Scale, subscale “psychological distress”; CC-MMDS-BS, Climate Change Version of the Man Made Disaster-Related Distress Scale, subscale “change of existing belief systems”. As the factorial structure of the CC-MMDS was modified in the present study, statistics are given for the original version (“OLD”) and for the version used in subsequent analyses in this publication.

For the eco-distress questionnaires and the mental health questionnaires, 2.5% of sum scores were missing. Due to the small percentage, we chose analysis-specific case-wise deletion. In our sample, *n* = 79 (39%) participants screened positive for a generalized anxiety disorder, and *n* = 93 (51%) of the sample screened positive for a depressive disorder. Applying the same criterion to the GAD-7-C, *n* = 12 (6%) screened positive for eco-distress which is equivalent in severity to the symptom load of a generalized anxiety disorder. The distribution of symptom severity is displayed in **Table 2**. For the EAQ-22 subscale explicitly assessing functional impairments *(“Negative Consequences of Eco-Anxiety”)*, n = 23 (11%) participants had a sum score ≥ 18 (Min = 9, Max = 36), equal to indicating on average “tend to agree” for all items.

**Table 2:** Severity of depressive and anxious symptoms and of anxious symptoms regarding climate change.

Questionnaire	Minimal	Mild	Moderate	Severe	Missing
PHQ-9^1^	28 (14%)	62 (31%)	54 (26%)	39 (19%)	20 (10%)
GAD-7	34 (17%)	86 (42%)	59 (29%)	20 (10%)	4 (2%)
GAD-7-C	157 (77%)	30 (15%)	11 (5%)	1 (1%)	4 (2%)

PHQ-9, Patient Health Questionnaire; GAD-7, Generalized Anxiety Disorder Scale; GAD-7-C, Generalized Anxiety Disorder Scale Climate Version.

^1^PHQ-9 scores were retrieved from the general psychometric assessment for participants who were at the beginning of their therapy. Not all data was retrievable, thus more values were missing. If symptom severity is at least moderate, a depressive or anxiety disorder is probable.

### 3.2 Psychometric qualities of EAQ-22, GAD-7-C, and CC-MMDS in a clinical sample

#### 3.2.1 Model testing

The Shapiro Wilk’s test normality test indicated that all questionnaires were not normally distributed (all *p* < 0.001), thus we report robust fit indices. Fit was good for the EAQ-22. For the GAD-7-C, fit was acceptable as the criterion was met for *CFI*, *TLI*, and *SRMR*, but not for the *RMSEA*. We still decided to keep the questionnaire in its current form as it represents a direct adaption of a well-established mental health questionnaire. For the CC-MMDS, however, model fit was not acceptable except for the *SRMR*. Thus, we decided to conduct an exploratory factor analysis to test for a better model fit with an adapted structure. The Kaiser-Meyer-Olkin test indicated excellent sampling adequacy (*KMO* = 0.95), and the Bartlett’s Test of Sphericity was significant, *X*² *(*120) = 2920.03, *p* <.001 (44). Scree plot and parallel analysis suggested two factors, thus an exploratory factor analysis with two factors was run. We chose promax-rotation because the factors in the original publication had been strongly correlated (*r* = 0.74; (33). In our model, four items showed cross-loadings > 0.30 and were removed. These items addressed an emotion regulation strategy (avoidance of the topic; item3), anger or rage as an emotional reaction toward climate change (item 8); fear of future negative consequences of climate change (item 15); and difficulties in positive outlook due to climate change (item 17). Moreover, item 10 (“The extent of climate change has shaken my worldview”) was re-allocated from the *Psychological Distress* to the *Change of Existing Belief Systems* factor. Model fit of the new model was tested running a CFA. Model fit was acceptable for the adapted version, with only the *RMSEA* not fully meeting the criterion. All fit indices are displayed in **Table 3**.

**Table 3:** Fit indices for confirmatory factor analyses for EAQ-22, GAD-7-C, and CC-MMDS.

Fit statistic: criterion:	CFI > 0.90	TLI > 0.90	RMSEA < 0.08	SRMR < 0.08
EAQ-22	0.927	0.919	0.066	0.064
GAD-7-C	0.957	0.935	0.116	0.039
CC-MMDS_OLD	0.891	0.873	0.123	0.066
CC-MMDS_NEW	0.955	0.944	0.091	0.048

EAQ-22, Eco-Anxiety Questionnaire; GAD-7-C, Generalized Anxiety Disorder Scale Climate Version; CC-MMDS_OLD, Original version of the Climate Change Version of the Man Made Disaster-Related Distress Scale as presented by (33); CC-MMDS_NEW, adapted version of the CC-MMDS showing a good fit in our sample.

Factor loadings for all items in the exploratory factor analysis and the new factorial structure can be found in **Supplementary File S1**. The factor *Psychological Distress* now consists of seven items and covers climate change-related feelings of anxiety, insecurity, depression, helplessness, and guilt, as well as climate change-related impairments in concentration and ability to focus one’s thoughts. The factor *Change of Existing Belief Systems* now consists of five items and covers doubts regarding the world, humanity, justice, political decisions, norms, and values when taking into account how human society reacts to climate change.

#### 3.2.2 Reliability and validity analyses for EAQ-22, GAD-7-C, and CC-MMDS

**Figure 2:**
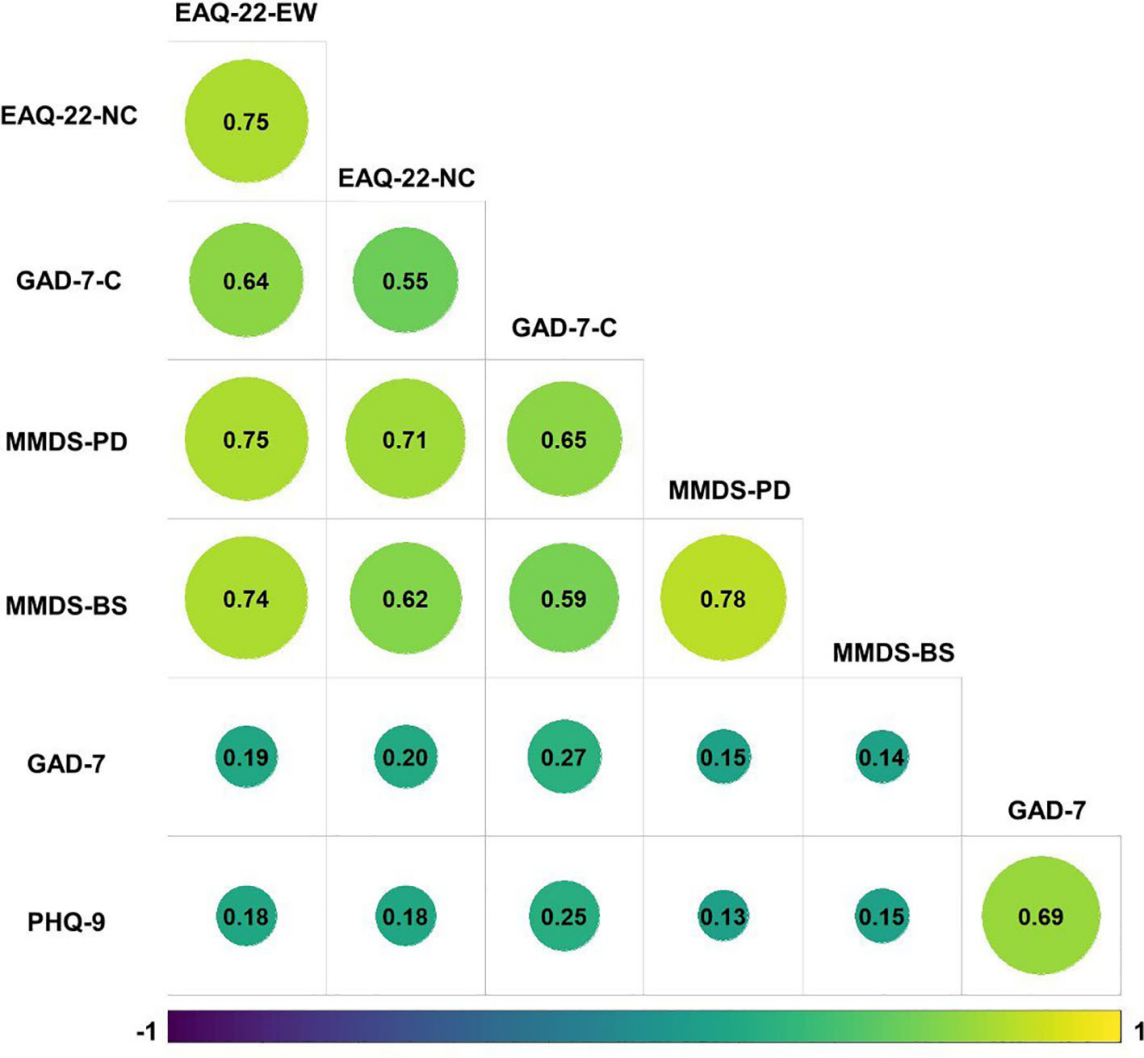
Spearman-rank correlations of EAQ-22, GAD-7-C, CC-MMDS, GAD-7, and PHQ-9. Size of the circle mirrors the size of the correlation, color indicates a negative or positive correlation. There are only positive correlations in this plot. EAQ-22-NC = Eco-Anxiety Questionnaire, subscale Negative Cognitions; EAQ-22-EW = Eco-Anxiety Questionnaire, subscale Habitual Ecological Worry; GAD-7-C = Generalized Anxiety Disorder Scale Climate Version; MMDS-PD = Climate Change Version of the Man Made Disaster-Related Distress Scale, subscale Psychological Distress; MMDS-BS = Climate Change Version of the Man Made Disaster-Related Distress Scale, subscale Change of Existing Belief Systems.

Reliability was assessed via Cronbach’s α as a measure of internal consistency. Reliability was high for the EAQ-22 (α = 0.94) and its subscales (EAQ-22-EW, α = 0.94; EAQ-22-NC, α = 0.98), for the CC-MMDS (α = 0.94) and its subscales (CC-MMDS-PD, α = 0.92; CC-MMDS-BS, α = 0.92), and for the GAD-7-C (α = 0.92). Correlations of the eco-distress questionnaires and questionnaires assessing general depressive and anxious symptoms are shown in **Figure 2**. We chose the Spearman coefficient because it has been shown to be more accurate if distributions are heavy-tailed or when outliers are present (De Winter et al., 2016), which was the case for our data. As hypothesized, there were high positive correlations between the different subscales of the eco-distress questionnaires (0.55 ≤ *r* ≤ 0.78) and small to moderate positive correlations between eco-distress questionnaires and general mental health questionnaires (0.13 ≤ *r* ≤ 0.27).

#### 3.2.3 Measurement invariance for EAQ-22, GAD-7-C, and CC-MMDS

Measurement Invariance was tested for gender and age. Gender was divided into male (n = 66) and female (*n* = 127), with *n* = 10 (4%) missing values. Age was divided into three groups of comparable size, namely participants younger than 30 years (*n* = 85), aged 30–45 years (*n* = 65), and older than 45 years (*n* = 51), with *n* = 2 (1%) missing values. For the EAQ-22, scalar invariance for both age and gender could be established. For the GAD-7-C, scalar invariance could be established for gender, and metric invariance for age. For the CC-MMDS, scalar invariance could be established for age, and metric invariance for gender. All fit indices are provided as part of **Supplementary File S1**.

### 3.3 Relationship of depressive and anxious symptoms with eco-distress

#### 3.3.1 Differences in scores on EAQ-22, GAD-7-C, and CC-MMDS for participants screening positive for a generalized anxiety disorder or a depressive disorder

**Figure 3:**
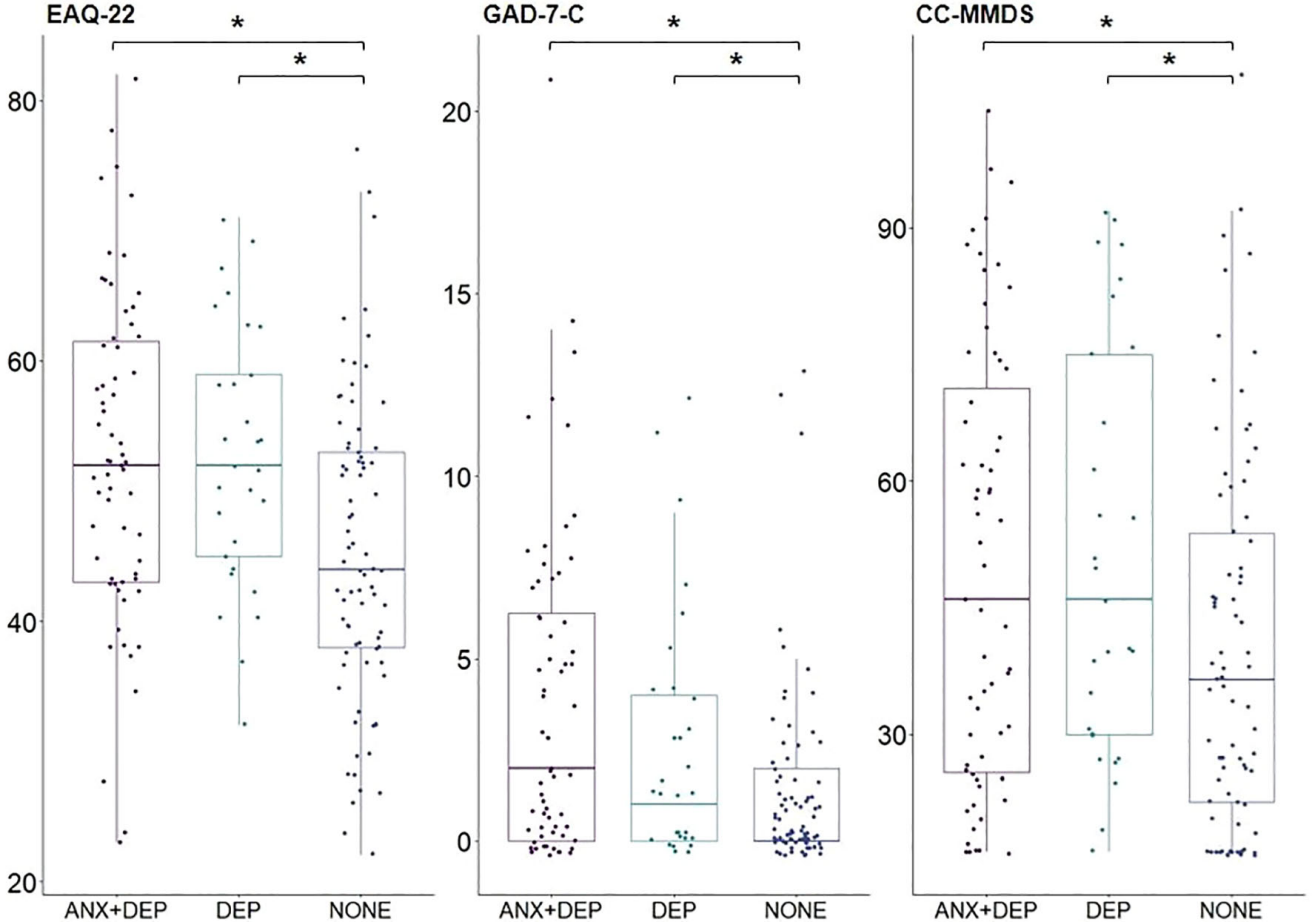
Comparison of symptom severity of EAQ-22, GAD-7-C, and CC-MMDS for patients with and without a positive screening for an anxious or depressive disorder. GAD + DEP = Positive screening for generalized anxiety disorder and depressive disorder; DEP = Positive screening for depressive disorder; NONE = negative screening for generalized anxiety disorder and depressive disorder. *p < 0.05.

To explore if scores of climate change distress differed depending on positive screening for a depressive or generalized anxiety disorder, we divided participants into four groups: positive screening for generalized anxiety disorder (*n* = 9), positive screening for depressive disorder (*n* = 30), both (*n* = 63), or none (*n* = 79). Group membership depended on GAD-7 scores and PHQ-9 scores. As there were only *n* = 9 participants who screened positive for a generalized anxiety disorder, but nor for a depressive disorder, we excluded them from the analysis due to small sample size. As the assumption of multivariate normality was violated for our data, we performed nonparametric multivariate model testing for EAQ-22, GAD-7-C, and CC-MMDS using the R package *npmv* (45). We report Wilks’ Lambda for the *F* approximation. Degrees of freedom and relative effects are provided in **Supplementary File S1**. For the EAQ-22, participants who screened positive for both an anxious and a depressive disorder and participants who screened positive for a depressive disorder showed significantly higher values than participants who screened negative for both disorders (*F* = 8.59, *p* < 0.001). The same pattern emerged for the GAD-7-C (*F* = 7.331, *p* = 0.001) and the CC-MMDS (*F* = 3.833, *p* = 0.024). Boxplots of scores per group and scale are shown in **Figure 3**.

#### 3.3.2 Prediction of change in EAQ-22, GAD-7-C, and CC-MMDS depending on change of depressive and anxious symptoms

Next, we explored the influence of depressive and anxious symptoms on symptoms of eco-distress over time, based on a second assessment of our sample. Of all study participants, *n* = 120 (59%) gave permission to contact them again after five months for a retest. Of these, *n* = 73 (59%) filled out the questionnaires at *t2*. We decided to refrain from conducting more elaborate analyses like path analysis or structural equation models due to the small sample size. We calculated the change scores ΔEAQ-22, ΔGAD-7-C, and ΔCC-MMDS and ran regression analyses with change scores ΔGAD-7 and ΔPHQ-9 as predictors. Sum scores at *t1* and *t2* correlated with *r* = 0.92 for EAQ-22, *r* = 0.70 for GAD-7-C, *r* = 0.80 for CC-MMDS, *r* = 0.60 for GAD-7, and *r* = 0.70 for PHQ-9. Multiple regression models were run for standardized variables. Homoscedasticity was checked visually via comparing residuals versus fitted values. No issues were detected. Autocorrelation of errors was tested via the Durbin-Watson test. All values were acceptable, with *DW* < 2.6 and no significant results (*p* > 0.05). Normality of residuals was tested with the Shapiro-Wilk test. All values were acceptable, with no significant results (*p* > 0.05). Multicollinearity was not an issue, with a variance inflation factor (VIF) < 1.5 across all analyses. Changes in severity of anxious or depressive symptoms were no significant predictors for symptom change on EAQ-22 and GAD-7-C. For the CC-MMDS, an increase of anxious symptoms and a decrease of depressive symptoms significantly predicted an increase of eco-distress, with *R^2^* = 0.082 and adjusted *R^2^* = 0.055. However, while ΔPHQ-9 and ΔGAD-7 correlated highly (*r* = 0.57), ΔCC-MMDS and ΔGAD-7 (*r* = 0.01) and ΔPHQ-9 (*r* = -0.17) correlated weakly. Moreover, in linear regression with only one of the two predictors, neither ΔGAD-7-C nor ΔPHQ-9 significantly predicted ΔCC-MMDS. Thus, a suppression effect was assumed, and the results should not be interpreted. Statistical results are provided in **Table 4**.

**Table 4:** Multiple regression analysis predicting ΔEAQ-22, ΔGAD-7-C, and ΔCC-MMDS with ΔGAD-7 and ΔPHQ-9 (n = 79). .

ΔEAQ-22				
Variable	β	SE_B_	T	P
Intercept	-0.007	0.119	-0.058	0.954
ΔGAD-7	0.172	0.145	1.180	0.242
ΔPHQ-9	0.018	0.147	0.123	0.903
*R^2^* = 0.034, adjusted *R^2^* = 0.005				
ΔGAD-7-C				
Variable	β	SE_B_	t	p
Intercept	-0.002	0.121	0.015	0.988
ΔGAD-7	-0.046	0.147	-0.312	0.756
ΔPHQ-9	-0.020	0.148	-0.135	0.893
*R^2^* = 0.004, adjusted *R^2^* = 0				
ΔCC-MMDS				
Variable	β	SE_B_	t	p
Intercept	0.001	0.116	0.011	0.991
ΔGAD-7	0.283	0.142	1.998	0.049
ΔPHQ-9	-0.330	0.143	-2.315	0.024
*R^2^* = 0.082, adjusted *R^2^* = 0.055				

ΔEAQ-22, Change in Eco-Anxiety Questionnaire from t1 to t2; ΔGAD-7-C, Change in Generalized Anxiety Disorder Scale Climate Version from t1 to t2; ΔCC-MMDS, Change in Climate Change Version of the Man Made Disaster-Related Distress Scale from t1 to t2; ΔGAD-7, Change in Generalized Anxiety Disorder Scale from t1 to t2; ΔPHQ-9, Change in Patient Health Questionnaire from t1 to t2.

### 3.4 Relationship of eco-distress, emotion regulation strategies, depressive and anxious symptoms

#### 3.4.1 Eco-distress and emotion regulation strategies

To assess which emotion regulation strategies might be relevant for the development and maintenance of eco-distress, we ran multiple regression analyses with the nine emotion regulation strategies we had adapted to climate change (CERQ-SC) as predictors for EAQ-22, GAD-7-C, and CC-MMDS. Again, we fitted a multiple regression model. Homoscedasticity was checked visually via comparing residuals versus fitted values. No issues were detected Autocorrelation of errors was tested via the Durbin-Watson test. All values were acceptable, with *DW* < 2.3 and no significant results (*p* > 0.05). Normality of residuals was tested with the Shapiro-Wilk test. Values were acceptable for the EAQ-22 and the CC-MMDS, with no significant results (*p* > 0.05). For the GAD-7-C, there were some outliers, with *W* = 0.87, *p* < 0.01. Multicollinearity was not an issue, with a VIF < 2.5 across all analyses. Results are displayed in **Table 5**. Higher scores for the emotion regulation strategies *Catastrophizing* (e.g. “I keep thinking about how horrible climate change is”) and *Rumination* (e.g. “I think about how I feel because of climate change”) significantly predicted higher scores on all three measures of eco-distress, *p* < 0.05. Furthermore, lower scores for *Putting into Perspective* (“I tell myself there are worse things in life”) significantly predicted higher scores on EAQ-22 and CC-MMDS, *p* < 0.05. Higher scores for *Positive Reappraisal* (“I think I can learn something from the situation”) significantly predicted higher scores on GAD-7-C, *p* < 0.05. Finally, higher scores on *Self-Blame* (“I feel that I am the one who is responsible for what has happened due to climate change”) and on *Refocusing on Planning* (“I think about how I can change the situation”) significantly predicted higher scores on CC-MMDS, *p* < 0.05.

**Table 5:** Multiple regression analysis predicting scores of EAQ-22, GAD-7-C, and CC-MMDS with scores on nine climate change-adapted emotion regulation strategies of the CERQ-Short.

EAQ-22				
Variable	β	SE_B_	T	P
Intercept	0.020	0.045	0.448	0.655
Self-blame	0.079	0.054	1.454	0.148
Acceptance	-0.014	0.049	-0.292	0.771
Rumination	**0.165**	**0.064**	**2.573**	**0.011**
Positive Refocusing	0.020	0.049	0.405	0.686
Planning	0.081	0.056	1.437	0.152
Positive Reappraisal	0.106	0.054	1.943	0.054
Perspective	**-0.222**	**0.051**	**-4.362**	**< 0.001**
Catastrophizing	**0.413**	**0.069**	**5.959**	**< 0.001**
Other Blame	0.074	0.053	1.381	0.169
*R^2^* = 0.620, *R^2adj^* = 0.601				
GAD-7-C				
Variable	β	SE_B_	t	p
Intercept	0.015	0.059	0.251	0.802
Self-blame	-0.046	0.072	-0.636	0.526
Acceptance	-0.126	0.065	-1.931	0.055
Rumination	**0.204**	**0.085**	**2.392**	**0.018**
Positive Refocusing	-0.064	0.064	-0.996	0.321
Planning	0.064	0.074	0.858	0.392
Positive Reappraisal	**0.170**	**0.072**	**2.355**	**0.020**
Perspective	0.086	0.067	1.285	0.201
Catastrophizing	**0.357**	**0.091**	**3.901**	**< 0.001**
Other Blame	0.001	0.070	0.012	0.991
*R^2^* = 0.357, *R^2adj^* = 0.326				
CC-MMDS				
Variable	β	SE_B_	t	p
Intercept	0.011	0.040	0.261	0.794
Self-blame	**0.105**	**0.048**	**2.163**	**0.032**
Acceptance	0.003	0.044	0.075	0.941
Rumination	**0.229**	**0.059**	**3.908**	**< 0.001**
Positive Refocusing	-0.056	0.044	-1.274	0.204
Planning	**0.147**	**0.052**	**2.845**	**0.005**
Positive Reappraisal	0.037	0.049	0.755	0.451
Perspective	**-0.092**	**0.045**	**-2.014**	**0.045**
Catastrophizing	**0.456**	**0.064**	**7.172**	**< 0.001**
Other Blame	0.062	0.048	1.279	0.202
*R^2^* = 0.693, *R^2adj^* = 0.678				

EAQ-22, Eco-Anxiety Questionnaire; GAD-7-C, Generalized Anxiety Disorder Scale Climate Version; CC-MMDS, Climate Change Version of the Man Made Disaster-Related Distress Scale. Bold values = significant at *p* < 0.05.

#### 3.4.2 Mediating effect of emotion regulation strategies on the relationship of depressive and anxious symptoms and eco-distress

**Figure 4:**
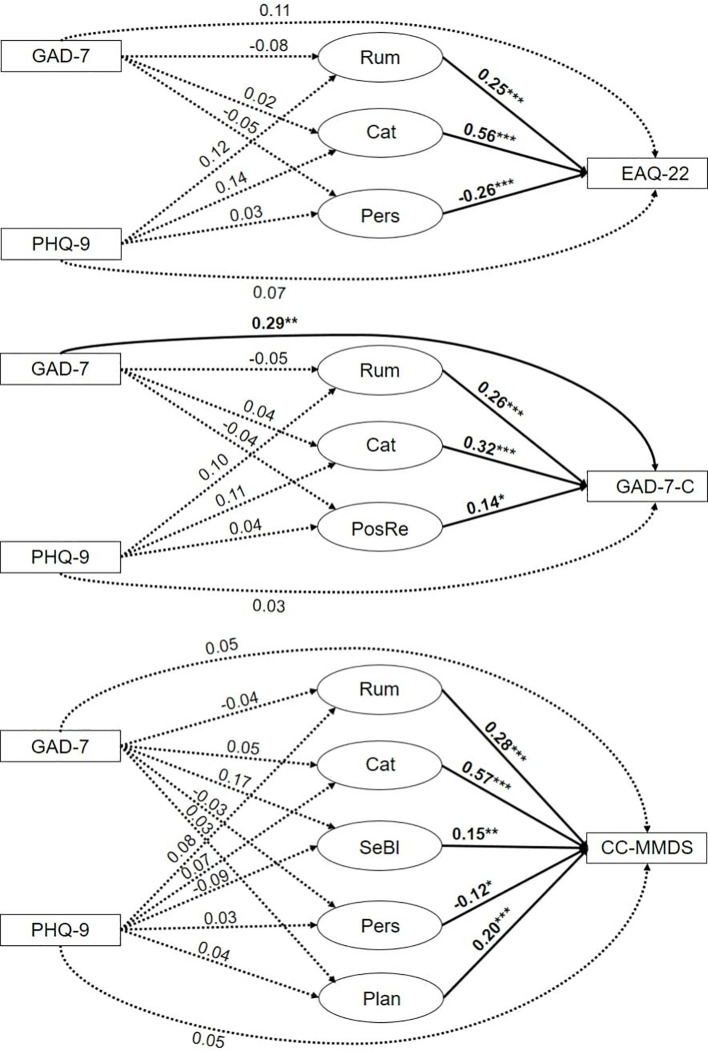
Mediation analysis with emotion regulation strategies Rumination (Rum), Catastrophizing (Cat), Positive Reappraisal (PosRe), Perspective (Pers), Self-Blame (SeBl), and Planning (Plan) as mediators for the relationship of anxious (GAD-7) and depressive (PHQ-9) symptoms with eco-distress (EAQ-22, GAD-7-C, CC-MMDS).

Lastly, we tested whether emotion regulation strategies would mediate the relationship of depressive and anxious symptoms with eco-distress, as would be expected of a variable representing the process through which two other variables are related. We ran separate mediation analyses for the three eco-distress questionnaires, including only emotion regulation strategies which significantly predicted eco-distress in a linear regression, as this a prerequisite for a variable to be a mediator. Through this design, we minimized the number of paths which had to be estimated. The mediation models are shown in **Figure 4**. As can be seen, only the direct effect of anxious symptoms on the GAD-7-C was significant, meaning that in all other cases, depressive and anxious symptoms did not significantly predict the level of eco-distress. Moreover, none of the indirect paths showed to be significant, as emotion regulation strategies significantly predicted eco-distress, but no emotion regulation strategy was significantly predicted by depressive or anxious symptoms.

## 4 Discussion

To explore the relationship of eco-distress with depressive and anxious symptoms and emotion regulation strategies in psychotherapy patients, we initially established the factorial validity of three eco-distress mental health questionnaires, namely the EAQ-22, the GAD-7-C, and the CC-MMDS, in a clinical population. While the EAQ-22 and GAD-7-C showed an acceptable fit, the factorial structure of the CC-MMDS could not be replicated, and we conducted an exploratory factor analysis to determine an adapted factorial structure with adequate model fit. For all questionnaires assessing eco-distress, participants who screened positive for a depressive disorder or both a depressive and a generalized anxiety disorder showed significantly higher values than participants who screened negative for both disorders. However, change in anxious or depressive symptoms did not predict a change in eco-distress when re-assessed after five months. Moreover, while several emotion regulation strategies significantly predicted the level of eco-distress experienced by participants, none significantly mediated the relationship of depressive and anxious symptoms with eco-distress.

The results of the model tests in our sample indicate that the EAQ-22, as well as the GAD-7-C, are well suited to evaluate eco-distress in a sample of psychotherapy out-patients. While the EAQ-22 offers the advantage of dividing symptoms of eco-distress into negative emotional reactions (*Habitual Ecological Worry*) and functional impairments (*Negative Consequences of Eco-Anxiety*), the GAD-7-C offers the possibility to compare the severity of symptomology with established cut-offs for generalized anxiety disorder. However, symptoms pertaining to a generalized anxiety disorder certainly only represent one component of eco-distress (6). For the CC-MMDS, future research would have to determine the psychometric quality of our adapted version in other samples. Item 10, “The extent of climate change has shaken my worldview”, seems to fit better with the factor *Change of Existing Belief Systems*, which it belongs to in our adapted version. Of note, it also pertained to this factor in the MMDS, the questionnaire assessing reactions to man-made disasters in general that the CC-MMDS is based on (34). Moreover, the assessment of changes in existing beliefs due to climate change is a unique feature among eco-distress questionnaires (46) and may be worth pursuing further.

We could replicate the moderate correlations of depressive and anxious symptoms with eco-distress reported for the general population (4, 14, 15). It is noteworthy that twelve participants (6%) screened positive for eco-distress which is equivalent in severity to the symptom load of a generalized anxiety disorder. In comparison, a representative survey of *n* = 1031 adults (> 18 years) from the US used the items of the PHQ-4 to assess eco-distress. The PHQ-4 is a shortened screener for depression and anxiety, employing the first two items of the GAD-7 and the PHQ-9 (47). In this survey, 1% of the sample indicated “nearly every day” and 2% “more than half of the days” for all PHQ-4 items, which equals a positive screening for depressive or anxious symptoms of significant severity (21). This underlines a heightened vulnerability toward the experience of eco-distress in persons with co-existing mental health impairments.

To further explore this heightened vulnerability, we tested whether the severity of eco-distress differed depending and anxious and depressive symptom severity. For all three questionnaires EAQ-22, GAD-7-C, and CC-MMDS, eco-distress was higher if participants screened positive for both a generalized anxiety disorder and a depressive disorder (ANX + DEP > NONE). There were no statistically significant differences between the two groups (ANX + DEP ≈ DEP). However, our results can only provide information about the correlation at the level of symptom severity assessed with self-report questionnaires, as the psychiatric diagnoses of the participants determined by clinicians were not part of our data set. While there seems to be a tendency for individuals with pronounced mental health impairments to report elevated levels of eco-distress, no association with a specific subset of depressive or anxious symptoms could be determined. This finding points toward trans-diagnostic factors, such as dysfunctional cognitive processes, which may cause elevated levels of distress in reaction to climate change as well as to other stimuli as a possible explanation for the heightened vulnerability of persons with mental health impairments (48, 49).

Emotion regulation strategies were evaluated as a possible dysfunctional cognitive process contributing to the level of eco-distress experienced by individuals. Indeed, *Rumination* and *Catastrophizing* explained a significant share of the variance for all three eco-distress questionnaires, *Putting into Perspective* for EAQ-22 and CC-MMDS, *Positive Reappraisal* for GAD-7-C, and *Self-Blame* and *Planning* for CC-MMDS. *Putting into Perspective* was the only emotion regulation strategy predicting lower scores of eco-distress. This result is in line with previous research showing that denial or shift of guilt, relativizing, and trusting that powerful others are in control can induce distancing effects which can mitigate distress (50–52). Perhaps counterintuitively, higher scores for *Positive Reappraisal* and for *Refocusing on Planning* predicted higher scores of eco-distress, as well. This finding suggests that the more time a person with mental health impairments thinks about climate change, the more prone that person is to experience higher levels of eco-distress. This might be linked to the fact that climate change is a problem which is unsolvable on the individual level, and therefore even typically proactive strategies might eventually lead to feelings of hopelessness and helplessness.

Overall, our results suggest that individuals with mental health impairments experience higher levels of eco-distress, even if they tend to employ adaptive emotion regulation strategies, while the only strategy correlated with lower eco-distress scores aims at emotional distancing from the climate crisis and its consequences. At a first glance, these findings might suggest that practitioners should indeed support individuals with mental health impairments in their efforts to distance themselves from their thoughts and feelings regarding the climate crisis. However, this assumption does not hold when considering its implications. For one, previous research shows that avoidance and eco-distress are positively correlated, as well (53, 54). Thus, if a person experiences eco-distress, ignoring these feelings might help to reduce them on a short-term basis – however, psychotherapy research clearly shows that avoiding or suppressing negative emotions are no viable long-term strategies (55). Secondly, in addition to the individual level, the implications of clinical decisions for the societal level have to be taken into account, as well. Eco-distress is strongly related to pro-environmental behavior (15, 56), and the emotions in itself are considered an adaptive response to a real threat (57). Therefore, the aim of interventions for individuals experiencing eco-distress cannot be to reduce these feelings to a minimum. Rather, individuals should be enabled to act on their feelings by reducing the functional impairments resulting from eco-distress which might hinder people from action and which present the main mental health burden. Indeed, a recent experimental study showed that moderate levels of eco-distress are associated with the highest level of pro-environmental behavior, while not being correlated with elevated levels of general anxiety and death anxiety (58). Developing a feeling of agency might be one possibility to support individuals in dealing with feelings of eco-distress in such an adaptive way: in an experimental study with young people, agency when faced with the climate crisis led to more meaning-focused coping and less anxiety (59).

Lastly, we tested whether emotion regulation strategies mediate the relationship of depressive and anxious symptoms with eco-distress. No mediation showed to be significant. Thus, while emotion regulation strategies explain a significant share of the variance in eco-distress, they do not offer an explanation for the heightened vulnerability of persons with co-existing mental health impairments. Moreover, the only significant direct effect in the mediation analyses was the prediction of GAD-7-C scores by GAD-7 scores, which could be expected due to the high similarity of the items. Therefore, our data shows a heightened vulnerability of individuals with co-existing mental health impairments; simultaneously, it shows that eco-distress is not a mere reflection of depressive and anxious symptoms. This finding is supported by our analysis of a re-assessment after five months, showing that none of the change scores for eco-distress were significantly predicted by change scores for anxious or depressive symptoms. Moreover, it is in line with a recent longitudinal study which showed that over time, climate change-related anger, fear, and sadness are distinct from general anger, fear, and sadness (60). However, as none of the emotion regulation strategies assessed in our study showed to be a significant mediator, it remains unknown which factors are causing the positive correlation of depressive and anxious symptoms with eco-distress and the heightened vulnerability of persons with mental health impairments.

Of note, emotion regulation strategies explained a large share of the overall variance in all three eco-distress questionnaires, ranging from 33% - 68%. Thus, emotion regulation strategies seem integral to the understanding of eco-distress, and a valuable target for interventions. In the treatment of depressive and anxiety disorders, targeted interventions to improve emotion regulation are well established and have been shown to be effective in reducing symptom severity (50, 61). Based on our results, an intervention targeted at building resilience against eco-distress would have to address the dysfunctional emotion regulation strategies of *Catastrophizing* and *Rumination.* One possibility would be to adapt successful interventions to the specificities of eco-distress. A recent randomized controlled trial employed a similar approach and adapted a trans-diagnostic treatment manual to eco-distress, with several modules addressing emotion regulation (62). Participants reported lower scores of eco-distress and of depressive symptoms. This further supports the hypothesis of shared dysfunctional cognitive processes which lead both to elevated levels of eco-distress and of general mental health impairments.

### 4.1 Limitations

Several limitations of our study have to be taken into account. Our sample partly consists of a convenience sample, and individuals who have a particular interest in climate change and its psychological consequences might have been more inclined to participate. Moreover, time in psychotherapy differed for our participants. Regarding participants’ mental health impairments, no conclusions regarding specific psychiatric diagnoses can be drawn from our data, as this information was not part of our data set. Our data conveys the effects of eco-distress in psychotherapy patients and the relationship with anxious and depressive symptom severity. While sampling psychotherapy out-patients allowed for the detailed analysis of the interplay of co-existing depressive and anxious symptoms and eco-distress, generalizability of our results to the general population is limited. Additionally, we could not explore the differences between participants screening positive for a generalized anxiety disorder with or without a positive screening for a comorbid depressive disorder, as only *n* = 9 (4%) of our sample screened positive for a generalized anxiety disorder, but not for a depressive disorder. However, the high comorbidity of depressive and anxious symptoms is common in a clinical sample (63) and mirrors the average symptom load of individuals with generalized anxiety disorder. Moreover, even though we aimed at collecting data at two time points, only a smaller fraction of our sample took part in the re-evaluation after five months, limiting the explanatory power of research mostly to cross-sectional data. Finally, the nature of our data does not allow conclusions on the effects of offering psychotherapy or counseling to individuals reporting elevated levels of eco-distress. While we discussed the implications of our findings on the interplay of eco-distress and emotion regulation strategies, future research would have to determine the actual effects of targeting these mechanisms in psychotherapy and counseling.

### 4.2 Conclusion

Eco-distress and co-existing mental health impairments are closely linked. Importantly, their positive correlation persists in a sample of individuals with pronounced depressive and anxious symptoms, and the prevalence of eco-distress is elevated in this population in comparison to the general population. Moreover, while previous research has established the central role of maladaptive emotion regulation strategies for the development of mental health impairments, our study elicited their role as a contributing factor to eco-distress, as well. Thus, targeting dysfunctional cognitive processes and maladaptive emotion regulation strategies related to eco-distress might prove a valuable objective for counseling and psychotherapy. Ultimately, these efforts might contribute to the psychological resilience of individuals faced with the adversity of climate change, thereby facilitating society’s efforts of climate change mitigation and adaption.

## Data Availability

The datasets presented in this study can be found in online repositories. The names of the repository/repositories and accession number(s) can be found below: https://osf.io/zgrqe/?view_only=d75a73f2e1b547aa8d1eeff00fc5323f.

## References

[1] Richardson K Steffen W Lucht W Bendtsen J Cornell SE Donges JF. Earth beyond six of nine planetary boundaries. Sci Adv. (2023) 9:eadh2458. doi: 10.1126/sciadv.adh2458, PMID: 37703365 PMC10499318

[2] Pörtner HO Roberts DC Adams H Adler C Aldunce P Ali E. Climate change 2022: Impacts, adaptation and vulnerability. IPCC Sixth Assess Rep. Cambridge, UK and New York, NY, USA: Cambridge University Press. (2022).

[3] Romanello M Walawender M Hsu SC Moskeland A Palmeiro-Silva Y Scamman D. The 2024 report of the Lancet Countdown on health and climate change: facing record-breaking threats from delayed action. Lancet. (2024) 404:1847–96. doi: 10.1016/S0140-6736(24)01822-1, PMID: 39488222 PMC7616816

[4] Boluda-Verdú I Senent-Valero M Casas-Escolano M Matijasevich A Pastor-Valero M. Fear for the future: Eco-anxiety and health implications, a systematic review. J Environ Psychol. (2022) 84:101904. doi: 10.1016/j.jenvp.2022.101904

[5] Clayton S Manning C Krygsman K Speiser M. Mental health and our Changing Climate: Impacts, Implications, and Guidance. Washington, DC: American Psychological Association (2017).

[6] Pihkala P. Toward a taxonomy of climate emotions. Front Clim. (2022) 3. doi: 10.3389/fclim.2021.738154

[7] Ágoston C Csaba B Nagy B Kőváry Z Dúll A Rácz J. Identifying types of eco-anxiety, eco-guilt, eco-grief, and eco-coping in a climate-sensitive population: A qualitative study. Int J Env Res Public Health. (2022) 19:2461. doi: 10.3390/ijerph19042461, PMID: 35206648 PMC8875433

[8] Hickman C Marks E Pihkala P Clayton S Lewandowski RE Mayall EE. Climate anxiety in children and young people and their beliefs about government responses to climate change: a global survey. Lancet Planet Health. (2021) 5:e863–73. doi: 10.1016/S2542-5196(21)00278-3, PMID: 34895496

[9] Van Valkengoed AM Steg L De Jonge P. Climate anxiety: A research agenda inspired by emotion research. Emot Rev. (2023) 15:258–62. doi: 10.1177/17540739231193752

[10] Coffey Y Bhullar N Durkin J Islam MS Usher K. Understanding eco-anxiety: A systematic scoping review of current literature and identified knowledge gaps. J Clim Change Health. (2021) 3:100047. doi: 10.1016/j.joclim.2021.100047

[11] Marks E Hickman C. Eco-distress is not a pathology, but it still hurts. Nat Ment Health. (2023) 1:379–80. doi: 10.1038/s44220-023-00075-3

[12] Brosch T. Affect and emotions as drivers of climate change perception and action: a review. Curr Opin Behav Sci. (2021) 42:15–21. doi: 10.1016/j.cobeha.2021.02.001

[13] Clayton S. Climate anxiety: Psychological responses to climate change. J Anxiety Disord. (2020) 74:102263. doi: 10.1016/j.janxdis.2020.102263, PMID: 32623280

[14] Gago T Sargisson RJ Milfont TL. A meta-analysis on the relationship between climate anxiety and wellbeing. J Environ Psychol. (2024) 94:102230. doi: 10.1016/j.jenvp.2024.102230

[15] Hogg TL Stanley SK O’Brien LV Watsford CR Walker I. Clarifying the nature of the association between eco-anxiety, wellbeing and pro-environmental behaviour. J Environ Psychol. (2024) 95:102249. doi: 10.1016/j.jenvp.2024.102249

[16] Kovács LN Jordan G Berglund F Holden B Niehoff E Pohl F. Acting as we feel: Which emotional responses to the climate crisis motivate climate action. J Environ Psychol. (2024) 96:102327. doi: 10.1016/j.jenvp.2024.102327

[17] American Psychiatric Association. Diagnostic and statistical manual of mental disorders, 5th. Washington, DC [u.a.]: American Psychiatric Assoc. (2013).

[18] Hohls JK König HH Quirke E Hajek A. Anxiety, depression and quality of life—a systematic review of evidence from longitudinal observational studies. Int J Environ Res Public Health. (2021) 18:12022. doi: 10.3390/ijerph182212022, PMID: 34831779 PMC8621394

[19] McKnight PE Monfort SS Kashdan TB Blalock DV Calton JM. Anxiety symptoms and functional impairment: A systematic review of the correlation between the two measures. Clin Psychol Rev. (2016) 45:115–30. doi: 10.1016/j.cpr.2015.10.005, PMID: 26953005

[20] Ramsay G Williams M Marks E Morgan G. A COSMIN systematic review of the psychometric properties of instruments that measure climate change-related distress. Cogent Ment Health. (2025) 4:1–27. doi: 10.1080/28324765.2025.2449878 PMC12443130 41262947

[21] Leiserowitz A Maibach E Rosenthal S Kotcher J Goddard E Carman J. Climate Change in the American Mind: Beliefs & Attitudes, Spring 2024. New Haven, CT: Yale Program on Climate Change Communication: Yale University and George Mason University (2024).

[22] Gebhardt N Schwaab L Friederich HC Nikendei C. The relationship of climate change awareness and psychopathology in persons with pre-existing mental health diagnoses. Front Psychiatry. (2023) 14:1274523. doi: 10.3389/fpsyt.2023.1274523, PMID: 38090707 PMC10715411

[23] Biggs A Brough P Drummond S. Lazarus and Folkman’s psychological stress and coping theory. The Handbook of Stress and Health. (2017) Wiley. 349–64. doi: 10.1002/9781118993811.ch21

[24] Wullenkord MC Johansson M Loy LS Menzel C Reese G. Go out or stress out? Exploring nature connectedness and cumulative stressors as resilience and vulnerability factors in different manifestations of climate anxiety. J Environ Psychol. (2024) 95:102278. doi: 10.1016/j.jenvp.2024.102278

[25] Ojala M. How do children cope with global climate change? Coping strategies, engagement, and well-being. J Environ Psychol. (2012) 32:225–33. doi: 10.1016/j.jenvp.2012.02.004

[26] Orrù L Taccini F Mannarini S. Worry about the future in the climate change emergency: A mediation analysis of the role of eco-anxiety and emotion regulation. Behav Sci. (2024) 14:255. doi: 10.3390/bs14030255, PMID: 38540558 PMC10967985

[27] von Elm E Altman DG Egger M Pocock SJ Gøtzsche PC Vandenbroucke JP. Strengthening the reporting of observational studies in epidemiology (STROBE) statement: guidelines for reporting observational studies. BMJ. (2007) 335:806. doi: 10.1136/bmj.39335.541782.AD, PMID: 17947786 PMC2034723

[28] Schauenburg H Dinger U Kriebel A Huber J Friederich HC Herzog W. Development of psychodynamic training institutes: Example of the Heidelberg Institute for Psychotherapy. Psychotherapeut. (2019) 64:46–54. doi: 10.1007/s00278-018-0320-2

[29] Ágoston C Urbán R Nagy B Csaba B Kőváry Z Kovács K. The psychological consequences of the ecological crisis: Three new questionnaires to assess eco-anxiety, eco-guilt, and ecological grief. Clim Risk Manag. (2022) 37:100441. doi: 10.1016/j.crm.2022.100441

[30] Zeier P Wessa M. Measuring eco-emotions: a German version of questionnaires on eco-guilt, ecological grief, and eco-anxiety. Discov Sustain. (2024) 5:29. doi: 10.1007/s43621-024-00209-2

[31] Schwaab L Gebhardt N Friederich HC Nikendei C. Climate change related depression, anxiety and stress symptoms perceived by medical students. Int J Environ Res Public Health. (2022) 19:9142. doi: 10.3390/ijerph19159142, PMID: 35897512 PMC9332784

[32] Spitzer RL Kroenke K Williams JBW Löwe B. A brief measure for assessing generalized anxiety disorder: the GAD-7. Arch Intern Med. (2006) 166:1092–7. doi: 10.1001/archinte.166.10.1092, PMID: 16717171

[33] Beckord J Krakowczyk JB Gebhardt N Geiser LS Kamler K Nikendei C. Development and validation of a climate change version of the man-made disaster-related distress scale (CC-MMDS). J Clim Change Health. (2024) 20:100356. doi: 10.1016/j.joclim.2024.100356 PMC12851373 41647124

[34] Krakowczyk JB Beckord J Planert J Kohl P Schweda A Teufel M. Development and psychometric evaluation of the Man-Made Disaster-Related Distress Scale (MMDS). Psychiatry Res. (2023) 324:115193. doi: 10.1016/j.psychres.2023.115193, PMID: 37062158

[35] Kroenke K Spitzer RL Williams JBW. The PHQ-9. J Gen Intern Med. (2001) 16:606–13. doi: 10.1046/j.1525-1497.2001.016009606.x, PMID: 11556941 PMC1495268

[36] Gross JJ. Emotion regulation: Taking stock and moving forward. Emotion. (2013) 13:359–65. doi: 10.1037/a0032135, PMID: 23527510

[37] Aldao A Nolen-Hoeksema S Schweizer S. Emotion-regulation strategies across psychopathology: A meta-analytic review. Clin Psychol Rev. (2010) 30:217–37. doi: 10.1016/j.cpr.2009.11.004, PMID: 20015584

[38] Garnefski N Kraaij V. Cognitive emotion regulation questionnaire – development of a short 18-item version (CERQ-short). Pers Individ Differ. (2006) 41:1045–53. doi: 10.1016/j.paid.2006.04.010

[39] Rosseel Y. lavaan: an R package for structural equation modeling. J Stat Softw. (2012) 48:1–36. doi: 10.18637/jss.v048.i02

[40] Hu L Bentler PM. Cutoff criteria for fit indexes in covariance structure analysis: Conventional criteria versus new alternatives. Struct Equ Model Multidiscip J. (1999) 6:1–55. doi: 10.1080/10705519909540118

[41] Tabachnick B Fidell L. Using Multivariate Statistics. 6th. Deutschland: Pearson (2013). Available online at: https://elibrary.pearson.de/book/99.150005/9781292034546 (Accessed April 23, 2025).

[42] Hirschfeld G Brachel RV. Improving Multiple-Group confirmatory factor analysis in R – A tutorial in measurement invariance with continuous and ordinal indicators. Pract Assess Res Eval. (2014) 19:1. doi: 10.7275/qazy-2946

[43] Putnick DL Bornstein MH. Measurement invariance conventions and reporting: The state of the art and future directions for psychological research. Dev Rev. (2016) 41:71–90. doi: 10.1016/j.dr.2016.06.004, PMID: 27942093 PMC5145197

[44] Williams B Onsman A Brown T. Exploratory factor analysis: A five-step guide for novices. Australas J Paramed. (2010) 8:1–13. doi: 10.33151/ajp.8.3.93

[45] Burchett WW Ellis AR Harrar SW Bathke AC. Nonparametric inference for multivariate data: the R package npmv. J Stat Softw. (2017) 76:1–18. doi: 10.18637/jss.v076.i04 36568334

[46] Van Dijk S Van Schie K Smeets T Mertens G. Limited consensus on what climate anxiety is: insights from content overlap analysis on 12 questionnaires. J Anxiety Disord. (2024) 109:102957. doi: 10.1016/j.janxdis.2024.102957, PMID: 39724678

[47] Löwe B Wahl I Rose M Spitzer C Glaesmer H Wingenfeld K. A 4-item measure of depression and anxiety: Validation and standardization of the Patient Health Questionnaire-4 (PHQ-4) in the general population. J Affect Disord. (2010) 122:86–95. doi: 10.1016/j.jad.2009.06.019, PMID: 19616305

[48] Foland-Ross LC Gotlib IH. Cognitive and neural aspects of information processing in major depressive disorder: an integrative perspective. Front Psychol. (2012) 3. doi: 10.3389/fpsyg.2012.00489, PMID: 23162521 PMC3495336

[49] Van Bockstaele B Verschuere B Tibboel H De Houwer J Crombez G Koster EHW. A review of current evidence for the causal impact of attentional bias on fear and anxiety. Psychol Bull. (2014) 140:682–721. doi: 10.1037/a0034834, PMID: 24188418

[50] Martin RC Dahlen ER. Cognitive emotion regulation in the prediction of depression, anxiety, stress, and anger. Pers Individ Differ. (2005) 39:1249–60. doi: 10.1016/j.paid.2005.06.004

[51] Bäuerle A Steinbach J Schweda A Beckord J Hetkamp M Weismüller B. Mental health burden of the COVID-19 outbreak in Germany: predictors of mental health impairment. J Prim Care Community Health. (2020) 11:2150132720953682. doi: 10.1177/2150132720953682, PMID: 32865107 PMC7457643

[52] Homburg A Stolberg A Wagner U. Coping with global environmental problems: development and first validation of scales. Environ Behav. (2007) 39:754–78. doi: 10.1177/0013916506297215

[53] Chapman DA Peters E. Examining the (non-linear) relationships between climate change anxiety, information seeking, and pro-environmental behavioral intentions. J Environ Psychol. (2024) 99:102440. doi: 10.1016/j.jenvp.2024.102440

[54] Wullenkord MC Tröger J Hamann KRS Loy LS Reese G. Anxiety and climate change: a validation of the Climate Anxiety Scale in a German-speaking quota sample and an investigation of psychological correlates. Clim Change. (2021) 168:20. doi: 10.1007/s10584-021-03234-6

[55] Lincoln TM Schulze L Renneberg B. The role of emotion regulation in the characterization, development and treatment of psychopathology. Nat Rev Psychol. (2022) 1:272–86. doi: 10.1038/s44159-022-00040-4

[56] Pavani JB Nicolas L Bonetto E. Eco-Anxiety motivates pro-environmental behaviors: A two-wave longitudinal study. Motiv Emot. (2023) 47:1062–74. doi: 10.1007/s11031-023-10038-x

[57] Clayton S. Climate change and mental health. Curr Env Health Rep. (2021) 8:1–6. doi: 10.1007/s40572-020-00303-3, PMID: 33389625

[58] Coates Z Brown S Kelly M. Understanding climate anxiety and potential impacts on pro-environment behaviours. J Anxiety Disord. (2025) 114:103049. doi: 10.1016/j.janxdis.2025.103049, PMID: 40540828

[59] Asbrand J Spirkl N Reese G Spangenberg L Shibata N Dippel N. Understanding coping with the climate crisis: an experimental study with young people on agency and mental health. Anxiety Stress Coping. (2024) 38:1–16. doi: 10.1080/10615806.2024.2388255, PMID: 39165166

[60] Contreras A Blanchard MA Mouguiama-Daouda C Heeren A. When eco-anger (but not eco-anxiety nor eco-sadness) makes you change! A temporal network approach to the emotional experience of climate change. J Anxiety Disord. (2024) 102:102822. doi: 10.1016/j.janxdis.2023.102822, PMID: 38159371

[61] Daros AR Haefner SA Asadi S Kazi S Rodak T Quilty LC. A meta-analysis of emotional regulation outcomes in psychological interventions for youth with depression and anxiety. Nat Hum Behav. (2021) 5:1443–57. doi: 10.1038/s41562-021-01191-9, PMID: 34545236 PMC7611874

[62] Lindhe N Bengtsson A Byggeth E Engström J Lundin M Ludvigsson M. Tailored internet-delivered cognitive behavioral therapy for individuals experiencing psychological distress associated with climate change: A pilot randomized controlled trial. Behav Res Ther. (2023) 171:104438. doi: 10.1016/j.brat.2023.104438, PMID: 38006766

[63] Simon NM. Generalized anxiety disorder and psychiatric comorbidities such as depression, bipolar disorder, and substance abuse. J Clin Psychiatry. (2009) 70:10–4. doi: 10.4088/JCP.s.7002.02, PMID: 19371501

