# Supplementary material for: Higher levels of eco-distress in psychotherapy out-patients with depressive and anxious symptoms are predicted by emotion regulation strategies
Source: Front Psychiatry. 2025 Oct 30;16:1664040. doi: 10.3389/fpsyt.2025.1664040 (PMC12612838; doi:10.3389/fpsyt.2025.1664040)
Supplement: Supplementary file 1 [file Supplementaryfile1.docx]

**Supplement S1**

**3.1 Sample Characteristics**


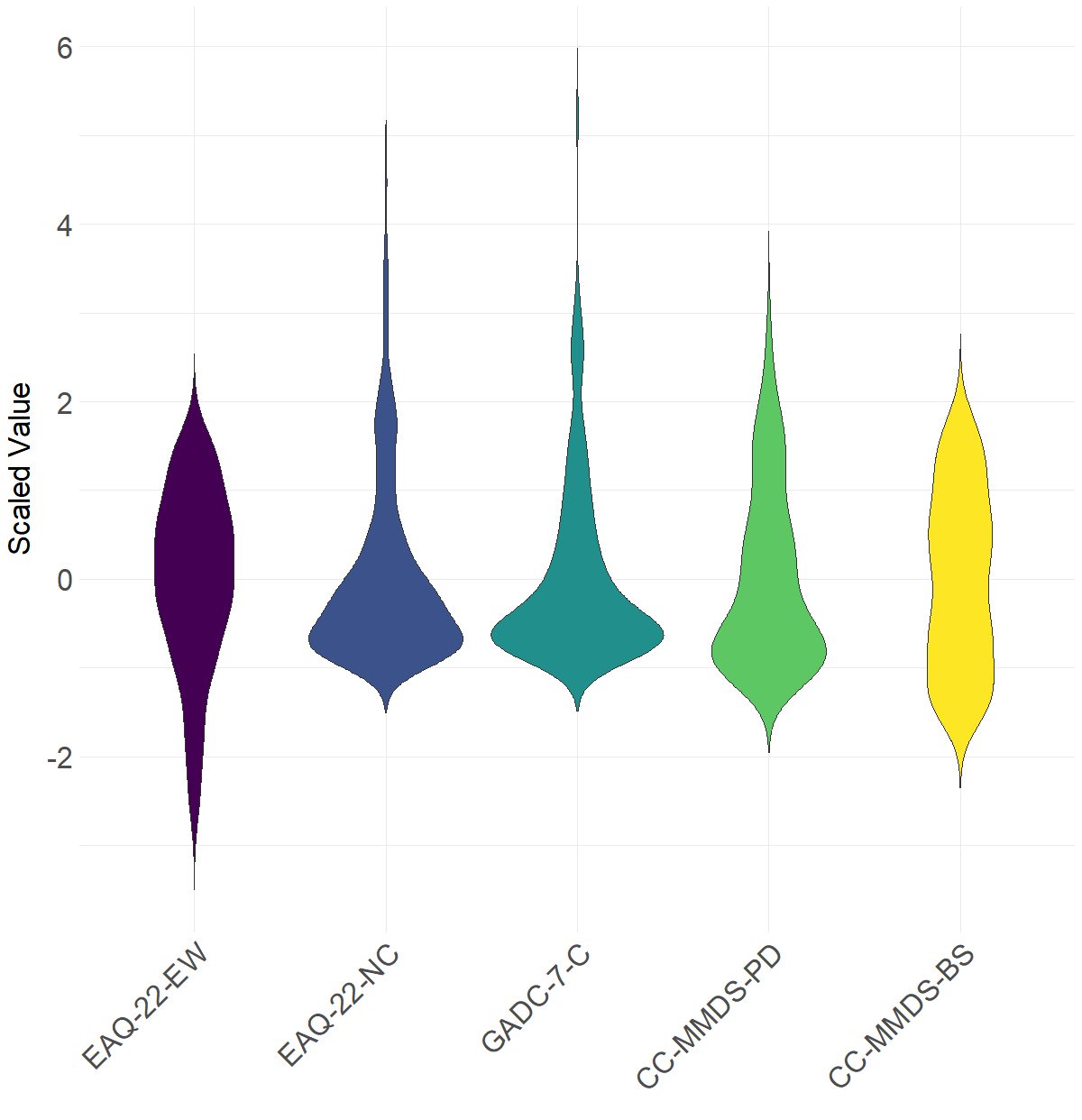


*Figure 1. Distribution of* z*-scaled means for GAD-7-C, EAQ-22, and CC-MMDS (in its adapted version)*

**3.2 Psychometric qualities of EAQ-22, GAD-7-C, and CC-MMDS in a clinical sample**

*Table 3. Factor loadings > 0.3 for all items included in the initial exploratory factor analysis of the CC-MMDS.*

| *Item^1^* | *Wording* |  | *Factor loadings on Psychological distress* | *Factor loadings on Change of existing belief systems* |
| --- | --- | --- | --- | --- |
| 01 | I had to think about the climate change, even without intending to. |  | 0.58 |  |
| 02 | I have felt anxious, worried, or nervous about the climate change. |  | 0.81 |  |
| 03 | I have distracted myself to avoid thinking about the climate change. |  | 0.92 | - 0.32 |
| 04 | I found it hard to concentrate when I thought about the climate change. |  | 0.86 |  |
| 05 | I felt depressed at the thought of climate change. |  | 0.78 |  |
| 06 | I felt helpless when I thought about the climate change. |  | 0.72 |  |
| 07 | I felt guilty when I thought about the climate change. |  | 0.78 |  |
| 08 | I felt anger or rage when I thought about the climate change. |  | 0.50 | 0.43 |
| 10 | The extent of the climate change has shaken my worldview. |  |  | 0.51 |
| 11 | The climate change made me doubt mankind. |  |  | 0.96 |
| 12 | The climate change made me doubt a just word. |  |  | 0.99 |
| 13 | I had my doubts about the political approach to climate change. |  |  | 0.70 |
| 14 | The climate change made me doubt social norms and values. |  |  | 0.88 |
| 15 | I was afraid of future negative consequences, which could be triggered by the climate change. |  | 0.48 | 0.44 |
| 16 | I felt more uncertainty than usual due to the climate change. |  | 0.65 |  |
| 17 | The climate change has made it increasingly difficult for me to look positively into the future. |  | 0.53 | 0.36 |

*^1^ Item No. 09 was part of the initial analysis in the construction of the CC-MMDS, but was removed by the authors during the factorial analysis. Thus, this item is missing.*


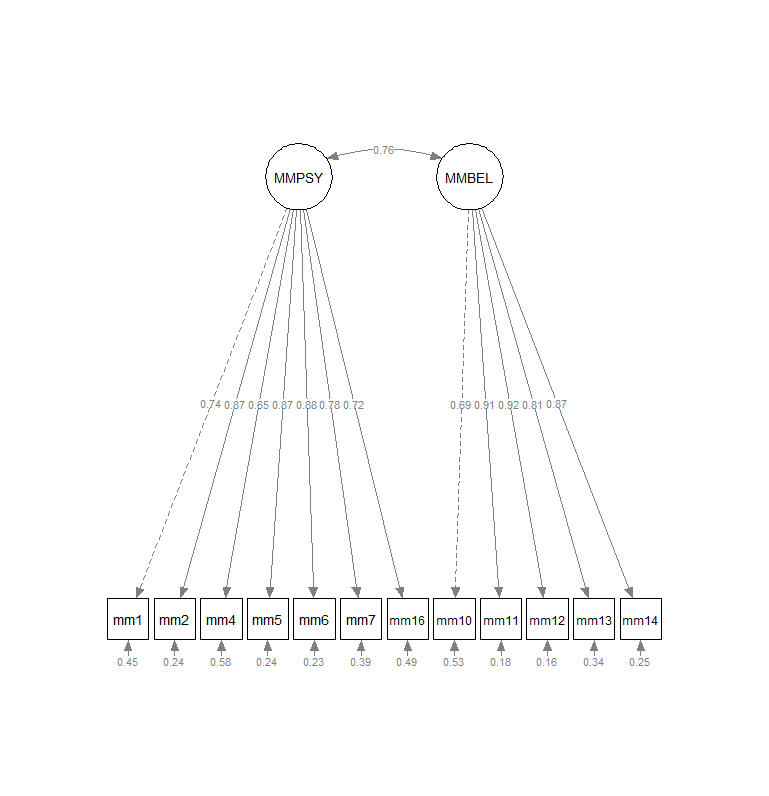


*Figure 2. Model structure of the revised version of the CC-MMDS. MMPSY = Psychological Distress, MMBEL = Change of Existing Belief Systems*

*Table 4. Results for measurement invariance regarding age and gender for EAQ-22, GAD-7-C, and CC-MMDS. Gender was divided into male (n = 66) and female (n = 127), with n = 10 (4%) missing values. Age was divided into three groups of participants < 30 years (n = 85), 30 – 45 years (n = 65), and > 45 years (n = 51) with n = 2 (1%) missing values. Configural, scalar, or metric invariance was presumed of ΔCFI < 0.1 and ΔRMSEA < 0.15 between models.*

| ***Measurement Invariance – EAQ-22 – Gender*** | | | | |
| --- | --- | --- | --- | --- |
| *Model* | *CFI* | *RMSEA* | *ΔCFI* | *ΔRMSEA* |
| Configural | 0.990 | 0.042 |  |  |
| Metric | 0.990 | 0.041 | 0 | -0.001 |
| **Scalar** | 0.989 | 0.041 | -0.001 | 0 |
| ***Measurement Invariance – EAQ-22 – Age*** | | | | |
| Configural | 0.991 | 0.038 |  |  |
| Metric | 0.985 | 0.048 | -0.006 | 0.010 |
| **Scalar** | 0.982 | 0.051 | -0.003 | -0.003 |
| ***Measurement Invariance – GAD-7-C – Gender*** | | | | |
| *Model* | *CFI* | *RMSEA* | *ΔCFI* | *ΔRMSEA* |
| Configural | 0.998 | 0.019 |  |  |
| Metric | 0.999 | 0.009 | 0.001 | 0.010 |
| **Scalar** | 1.000 | 0.000 | 0.001 | 0.009 |
| ***Measurement Invariance – GAD-7-C – Age*** | | | | |
| Configural | 0.971 | 0.094 |  |  |
| **Metric** | 0.977 | 0.074 | 0.006 | 0.020 |
| Scalar | 0.971 | 0.070 | -0.006 | -0.004 |
| ***Measurement Invariance – CC-MMDS – Gender*** | | | | |
| *Model* | *CFI* | *RMSEA* | *ΔCFI* | *ΔRMSEA* |
| Configural | 1.000 | 0.039 |  |  |
| Metric | 1.000 | 0.034 | 0 | -0.005 |
| **Scalar** | 1.000 | 0.030 | 0 | -0.004 |
| ***Measurement Invariance – CC-MMDS – Age*** | | | | |
| Configural | 0.997 | 0.038 |  |  |
| **Metric** | 0.989 | 0.066 | -0.008 | 0.028 |
| Scalar | 0.989 | 0.062 | 0 | -0.004 |

*
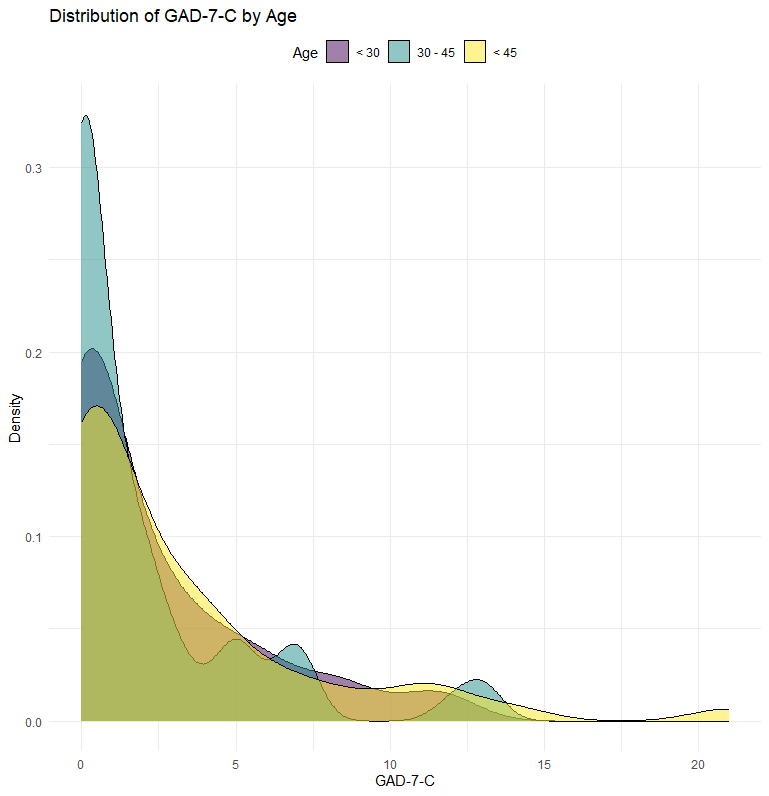
*

*Figure 3. Distribution of sum scores on GAD-7-C split by age.*


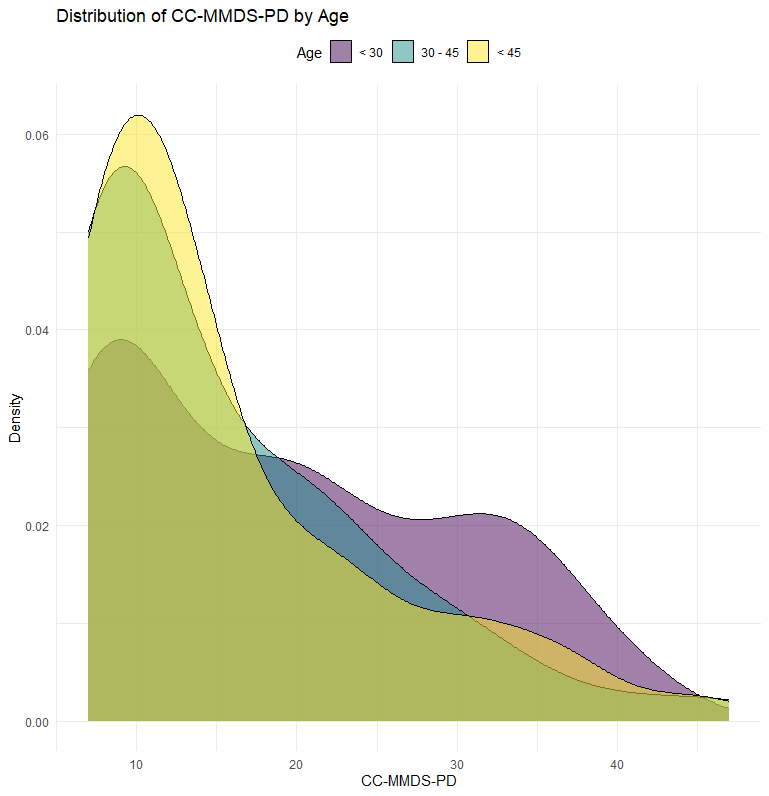


*Figure 4. Distribution of sum scores on CC-MMDS-PD split by age.*

*
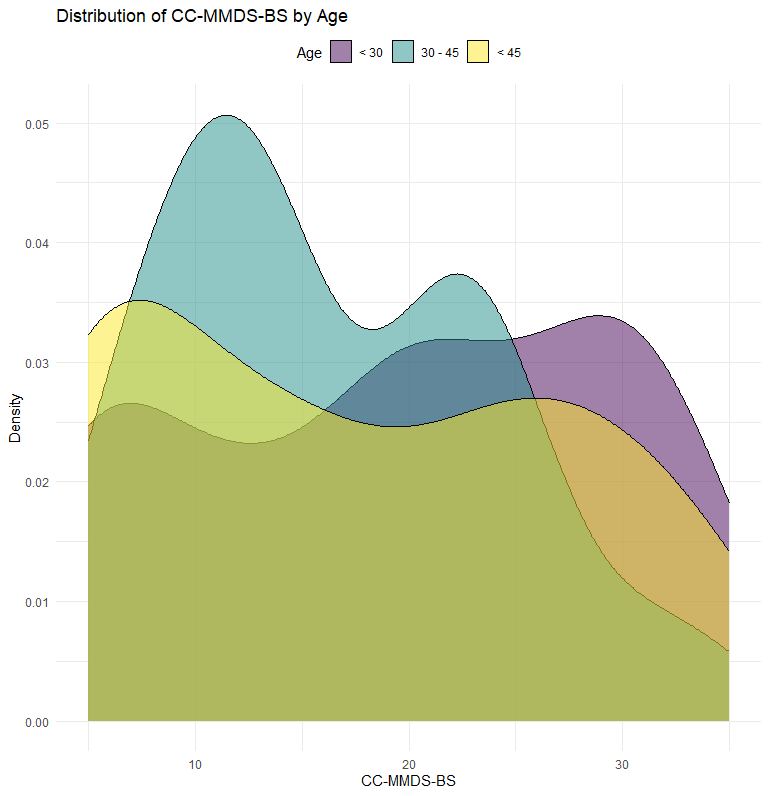
*

*Figure 5. Distribution of sum scores on CC-MMDS-BS split by age.*

**3.3 Relationship of Depressive and Anxious Symptoms with Eco-Distress**

*Table 5. Results for nonparametric multivariate model testing comparing scores for EAQ-22, GAD-7-C, and CC-MMDS across participants who screened positive for both a depressive and a generalized anxiety disorder (ANX + DEP), for a depressive disorder (DEP), or for none (NONE). Participants who screened positive for a generalized anxiety disorder, but negative for a depressive disorder, had to be excluded due to small sample size (n = 9). Relative effects refer to estimated difference in the central tendency of groups.*

| ***Questionnaires*** | ***Test Statistics*** | | | | ***Relative Effects*** | | |
| --- | --- | --- | --- | --- | --- | --- | --- |
|  | *F* | *df1* | *df2* | *p* | *ANX+DEP* | *DEP* | *NONE* |
| EAQ-22 | 8.594 | 2 | 168 | < 0.001 | 0.579 | 0.588 | 0.405 |
| GAD-7-C | 7.331 | 2 | 168 | 0.001 | 0.591 | 0.517 | 0.419 |
| CC-MMDS | 3.833 | 2 | 167 | 0.024 | 0.543 | 0.579 | 0.436 |

*Abbreviations: EAQ-22 = Eco-Anxiety Questionnaire*; *GAD-7-C = Generalized Anxiety Disorder Scale Climate Version; CC-MMDS = Climate Change Version of the Man Made Disaster-Related Distress Scale*
